# Prevalence of self-reported pain in patients 5 years after rectal cancer treatment: results from the prospective QoLiRECT cohort study

**DOI:** 10.2340/ao.v65.45198

**Published:** 2026-04-22

**Authors:** Tekla K. Nätterdal, Rode Grönkvist, Eva Haglind, Jacob Rosenberg, Elisabeth González, Dan Asplund, Sofie Walming, Eva Angenete

**Affiliations:** a Department of Surgery, Region Västra Götaland, Alingsås Lasarett, Alingsås, Sweden; b Department of Surgery, SSORG-Scandinavian Surgiacal Outcomes Reserch Group, Institute of Clinical Sciences, Sahlgrenska Academy, University of Gothenburg, Gothenburg, Sweden; c Biostatics, School of Public Health and Community Medicine, Institute of Medicine, Sahlgrenska Academy, University of Gothenburg, Gothenburg, Sweden; d Department of Surgery, Region Västra Götaland, Sahlgrenska University Hospital/Östra, Gothenburg, Sweden; e Department of Surgery, Herlev Hospital, University of Copenhagen, Herlev, Denmark; f Department of Surgery, Helsingborg Hospital, Helsingborg, Sweden

**Keywords:** colorectal neoplasms, treatment outcome, survivors, pain, prevalence, quality of life

## Abstract

**Background and purpose:**

Pain as a long-term outcome following rectal cancer treatment has received limited attention in previous studies. This prospective cohort study examined pain prevalence up to 5 years post-surgery in patients treated with curative intent. Secondary aims included evaluating pain intensity and its interference with daily life, and comparing pain experiences with a reference population. In addition, potential risk factors for pain were explored.

**Patient/material and methods:**

The study is based on the multicenter prospective cohort study QoLiRECT including patients from 16 surgical departments in Denmark and Sweden (2012–2015). Questionnaires regarding Quality of Life were administered at diagnosis and at 1, 2 and 5 years after treatment. Information about self-assessed pain was primarily derived from the ‘*Brief Pain Inventory – Short Form’*. Comparisons were made with an age- and sex- matched reference population.

**Results:**

Five years post-treatment, 37% of rectal cancer survivors reported pain exceeding common types of pain, with an average pain level of 3.8 and interference score of 3.1 (range 0–10). Pain was mainly located to the lower extremities, back and pelvis. Surprisingly, pain prevalence was higher in the reference population (53%), a difference consistent at all time points. No significant difference was found between groups regarding average pain or interference. Higher pain levels were associated with female sex, severe baseline pain, and elevated Body Mass Index (BMI).

**Interpretation:**

Pain was more common in the reference population, while intensity and interference were similar between groups. This suggests that the experience of pain may differ after surviving cancer, and such differences might be considered when studying pain in cancer survivors.

## Introduction

Colorectal cancer is the third most prevalent cancer worldwide with about 10% of all cancer diagnoses, with rectal cancer representing about 3.8% [1]. Over the past decades, advances in the treatment for rectal cancer have led to fewer local recurrences and improved survival rates [2, 3]. As more patients survive cancer, evaluating long-term outcomes such as functional impairments and quality of life (QoL) is becoming increasingly important [4]. One long-term outcome after cancer treatment is the presence of pain. Overall pain prevalence among patients previously treated for colorectal cancer has been estimated at 23% [5], and major and chronic pain has been described to be 17–31% [6, 7]. A cross-sectional study in patients treated with abdominoperineal excision (APE) found persistent perineal symptoms in 50% of all patients of whom 24% described symptoms of severe intensity [8].

Cancer-related pain may result from tissue damage caused by the tumor itself [9, 10], but may also be aggravated by (chemo)radiotherapy inducing, for example, neuropathic pain or insufficiency fractures [11–13] or surgery causing nerve and tissue damage [14]. Pretreatment characteristics, such as prior pain experiences and other comorbidities, may also affect pain severity after treatment [15].

Bowel, urinary, and sexual dysfunctions after treatment for rectal cancer have been quite well described [16–18] indicating effects on QoL and long-term survivorship [19–22]. However, only a limited number of studies have explored pain after treatment for rectal cancer [6, 7, 23]. These studies indicate that at least one third of patients experience pain in the lower extremities, and an association between pain and reduced QoL has been reported [7, 23]. Possible risk factors seem to be type of surgery, female sex, younger age, (chemo)radiotherapy and low income [7, 23, 24].

The primary aim of this study was to investigate the prevalence of pain during the last week 5 years after curative treatment for rectal cancer. Secondary aims were to investigate the prevalence of pain after 1 and 2 years, the level of average pain and its interference with daily life at every time point. We also wanted to explore the locations of pain and the number of pain sites in the body. Finally, we aimed to compare the outcomes to a reference population as well as explore potential risk factors for pain.

## Patients/material and methods

This study is based on the multicenter prospective cohort study QoLiRECT [25], a detailed QoL study that included patients who underwent treatment for rectal cancer between 2012 and 2015 at 16 surgical departments in Sweden and Denmark. Patients were offered participation when they had received information about their diagnosis and treatment and all patients signed informed consent. As previously reported, patients included in QoLiRECT study were somewhat younger with a lower tumor stage and with less comorbidity than patients who were not included [26]. The comprehensive and validated questionnaires assessed QoL and functional impairment, and were administered at diagnosis (baseline) and at 1, 2, and 5 years post-treatment [25]. Questionnaires were sent to the patients at home to reduce the risk of influences of health care personnel.

All patients with biopsy-verified rectal adenocarcinoma, regardless of tumor stage or intended treatment, were evaluated for inclusion in the QoLiRECT study. Exclusion criteria were age below 18 years and inability to understand and answer the questionnaire [25]. For this specific analysis (to reduce bias by direct pain caused by the rectal cancer itself), only patients undergoing treatment with a curative intent were eligible for inclusion. All patients who answered at least one questionnaire at any time point were included.

The questionnaire was constructed based on in-depth interviews with patients at appropriate time points along the rectal cancer trajectory to identify themes. Questions were constructed from themes and, together with questions on demographic details such as marital status, level of education, employment status, comorbidities, depression, alcohol consumption (using the AUDIT-C short form [27]), physical activity (measured using the Saltin-Grimby scale [28]), and BMI constituted the questionnaire. This was validated by an expert group consisting of surgeons, oncologists, anesthesiologists, and nurses and face-validated by appropriate patients who had not participated in the in-depth interviews [25].

The clinimetric approach was predominantly used [29], but some psychometrically validated questionnaires were also included, such as the Sense of Coherence scale [30] and EuroQol 5 Dimensions (EQ-5D) [31]. In the follow-up questionnaires a modified version of the ‘*Brief Pain Inventory’* was added, the ‘*Brief Pain Inventory – Short form’ (BPI-SF).* The BPI-SF is a validated questionnaire assessing self-reported pain regarding both severity and interference with daily life with a recall period of 1 week [32]. Unfortunately, this measuring tool was not included in the baseline questionnaire. Baseline data on pain were collected using the pain item from the EQ-5D (where recall period was the day patients answered the questionnaire), along with a few general pain-related questions.

Clinical information and details on tumor characteristics, as well as treatment, were collected from the Swedish Colorectal Cancer Registry and the Danish Colorectal Cancer Group Database [33, 34].

A reference sample of 3000 representative inhabitants of Sweden was retrieved from the Swedish Tax Authority, consisting of 250 men and 250 women per decade of age from 30 up to 89. They were contacted between June 2014 and November 2015 and invited to complete a similar questionnaire to that used in the QoLiRECT study, including the BPI-SF. A detailed description, as well as most demographic data, has been published elsewhere [35]. This age and sex matched cohort was used as a reference population for comparison regarding pain-related outcomes.

The prevalence of pain was derived from the modified BPI-SF, measuring pain exceeding common everyday kinds of pain. The interpretation guide of the BPI-SF was used for analysis of the questionnaire. However, in the BPI-SF there is a question consisting of a body diagram, a manikin drawing, in which patients can mark the localization of the affirmed pain. In cases where no answer was given to the optional screening question regarding experienced pain exceeding common types of pain, but the body diagram was filled in or the question regarding average pain was answered, we chose to interpret this as an indication of clinically relevant pain. Participants who completed the QoLiRECT questionnaire but skipped all questions in the modified BPI-SF, as well as skipped marking on the manikin, were in our analyses considered to be missing. Participants who responded ‘no’ to the screening question or ‘zero’ to the question regarding average pain were considered not to have pain beyond what is commonly experienced.

The amount of interference with daily life by pain was, according to *The Brief Pain Inventory User Guide*, scored as one of the following seven interference items: walking, work, mood, enjoyment of life, relations with others, sleep or general activity. These items address both activity-related and affective dimensions but were considered equivalent in terms of assessing their impact on daily life. Each item was answered by an 11-point Likert scale, and the average of the seven interference items were used if at least four items were answered.

A statistical analysis plan was detailed before the analyses were started. In the plan potential risk factors for pain were identified using a direct acyclic graph. Response rate was calculated removing recurrence/death as possible responders. Results regarding prevalence and interference of pain were described visually using bar charts and histograms and numerically using summary statistics (mean, median and quartiles). For comparison with the reference population, a subsample matched for age and sex was produced from the reference sample using the ‘Matching’ R package [36] with inverse variance matching. Comparisons to the matched reference sample were performed visually and with Brunner-Munzel tests [37] (for average pain and number of pain sites) and *t*-tests (for pain interference). *P* < 0.05 was considered statistically significant.

For the risk factor analysis, a linear mixed model for repeated measures was used, with average pain as reported in the BPI-SF [32] as the outcome. The *mixed model repeated measures* (MMRM) package of the R software was used to fit the model [38]*,* using the Satterthwaite approximation for effective degrees of freedom. An Autoregressive (AR) (1) covariance structure was specified and *restricted maximum likelihood estimation* (REML) was used.

As the primary aim of the study was descriptive, no imputation of missing data was performed. For risk factor analyses, the use of REML means inferences in the presence of missing data are valid under the assumption that data are missing at random, similar to common imputation methods.

## Results

**Figure 1:**
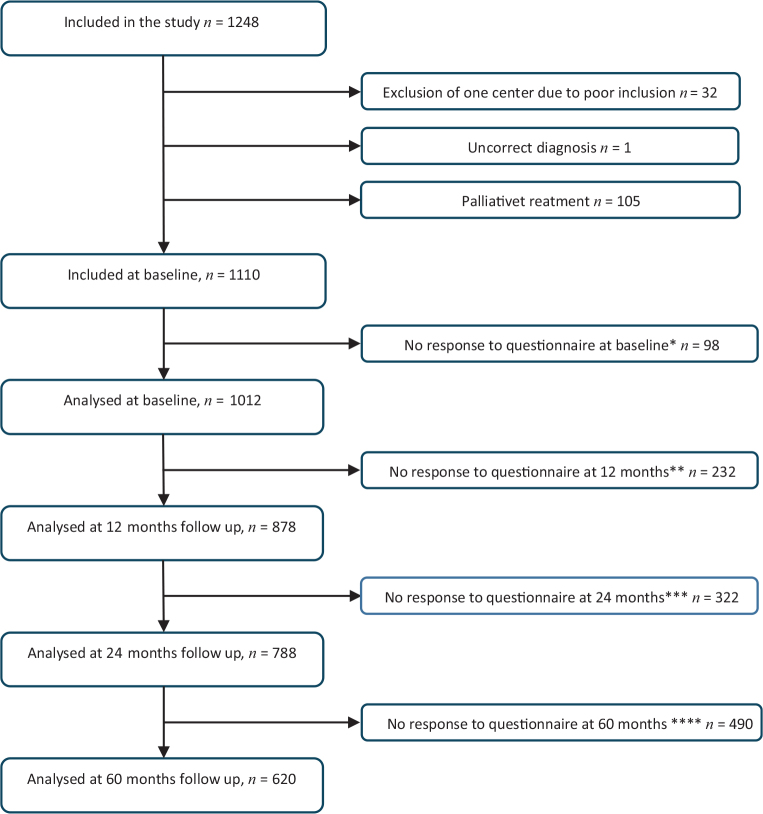
Flow chart. Reason for not responding: *at baseline (Not interested *n* = 70, Mixed/unkown *n* = 6, Not available *n* = 22) Response rate: 91%. **at 12 months (Not interested *n* = 70, Mixed/unkown = 65, Too ill *n* = 15, not available *n* = 54, recurrence/death *n* = 28) Response rate = 81%. ***at 24 months (No interested *n* = 70, mixed/unkown *n* = 97, Too ill *n* = 25, Not available *n* = 42, recurrence/death *n* = 88) Response rate = 78%. ****at 60 months (Not Interested *n* = 70, Mixed/unkown *n* = 154, Too ill *n* = 34, Not available *n* = 37, recurrence/death *n* = 195) Response rate = 68%. At baseline 1110 patients were included in the study. Of these, 70 participants never responded to any questionnaire, and a total of 1040 participants were analyzed. Not all patients answered all questionnaires.

A total of 1248 patients were recruited from 16 different surgical departments in Sweden and Denmark for the QoLiRECT study. After exclusion of one surgical department due to poor inclusion, and patients treated without curative intent, a total of 1110 patients were found eligible for inclusion in the study (Flow-chart, Figure 1). Seventy (6.3%) patients did not respond to any of the questionnaires at any time point, which left a sample of 1040 individuals. Response rates were calculated excluding patients with recurrence or death as possible responders. At baseline (i.e. when the questionnaire was administered after diagnosis), and at 1, 2, and 5 years, the response rates were 91, 81, 78 and 68%, respectively. At the 5-year follow-up, 195 (17.6%) patients had either died or experienced a recurrence of their rectal cancer. The reasons for not returning the questionnaire are presented in the flow-chart (Figure 1).

The demography for both the QoLiRECT cohort and the reference population is presented in Tables 1 and 2. Most patients were retired, were in a relationship, and a minority had a university education. A smaller part of the cohort was physically active (level 3–4 on the Saltin-Grimby Physical Activity Level Scale [28]), and about half of the participants had a prevalence of pain or discomfort at baseline according to the pain item in the EQ-5D; severe pain was prevalent in 2.6% at baseline. There was a higher proportion of men (62.8%), median age was 69 years (range 25–92) and median BMI 25.4 (range 15.5–55.5).

**Table 1:** Demographic and health-related characteristics

Patients (N)	Overall (*N* = 1040)	Missing
**Age**		
Median (Min, Max)	69.0 (25.0, 92.0)	
**Sex**		
Women	387 (37.2%)	0
Men	653 (62.8%)	
**BMI**		49 (4.7%)
Median (Min, Max)	25.4 (15.5, 55.5)	
**Type of surgical procedure**		66 (6.3%)
Anterior resection	518 (49.8%)	
Adominoperineal excision	340 (32.7%)	
Hartmann’s procedure	88 (8.5%)	
Other	28 (2.7%)	
**Neoadjuvant treatment**		32 (3.1%)
No	407 (39.1%)	
Preoperative radiotherapy	383 (36.8%)	
Preoperative chemotherapy/Chemoradiotherapy	218 (21.0%)	
**Adjuvant chemotherapy**		213 (20.5%)
No	493 (47.4%)	
Yes	334 (32.1%)	
UICC stage*****		67 (6.4%)
UICC 0	19 (1.8%)	
UICC I	295 (28.4%)	
UICC II	239 (23.0%)	
UICC III	331 (31.8%)	
UICC IV	89 (8.6%)	
**Self-reported depression**		36 (3.5%)
No	839 (80.7%)	
Yes	54 (5.2%)	
Don’t know	111 (10.7%)	
**In a relationship**		98 (9.4%)
Yes	702 (67.5%)	
No	240 (23.1%)	
**Level of education**		41 (3.9%)
No university education	791 (76.1%)	
University education	208 (20.0%)	
**Employment status**		39 (3.8%)
Employed	303 (29.1%)	
Retired	591 (56.8%)	
Other	107 (10.3%)	
Risk consumption of alcohol*		302 (29.0%)
No	623 (59.9%)	
Yes	115 (11.1%)	
Physical activity (according to Saltin-Grimby)**		54 (5.2%)
Saltin-Grimby 1–2	811 (78.0%)	
Saltin-Grimby 3–4	175 (16.8%)	
Sense of coherence (SOC-29)***		54 (5.2%)
Mean (SD)	159 (19.6)	
Median [Min, Max]	161 [85, 203]	
ASA classification****		74 (7.1%)
1	234 (22.5%)	
2	578 (55.6%)	
4–5	154 (14.8%)	
**Pain or discomfort at diagnosis (EQ5D)**		44 (4.2%)
None	513 (49.3%)	
Moderate	456 (43.8%)	
Severe	27 (2.6%)	
**Abdominal pain last month (at diagnosis)**		43 (4.1%)
No	683 (65.7%)	
Yes	314 (30.2%)	

* Alcohol Use Disorders Identification Test; Audit–C (risk consumption = women > 4, men > 5), [24].

** Saltin-Grimby scale, [25].

*** Sense of coherence scale score (SOC-29) [1].

**** ASA – American Association of Anesthesiology (ASA) physical status classification system.

***** Union for International Cancer Control

**Table 2:** Demography of the reference population.

Patients (N)	Overall (*N* = 620)	Missing
**Age**		0
Median (Min, Max)	67 (31, 90)	
**Sex**		0
Women	238 (38.4%)	
Men	382 (61.6%)	
**BMI**		15 (2.4%)
Median (Min, Max)	25.7 (16.7, 49.3)	
**In a relationship**		6 (1.0%)
Yes	483 (77.9%)	
No	131 (21.1%)	
**Level of education**		15 (2.4%)
No university education	434 (70.0%)	
University education	171 (27.6%)	
**Employment status**		1 (0.2%)
Employed	228 (36.8%)	
Retired	385 (62.1%)	
Other	6 (1.0%)	
Risk consumption of alcohol*		103 (16.6%)
No	388 (62.6%)	
Yes	129 (20.8%)	
Physical activity (Saltin-Grimby)**		3 (0.5%)
Saltin-Grimby 1–2	78 (12.6%)	
Saltin-Grimby 3–4	539 (86.9%)	
**Pain or discomfort at baseline (EQ5D)**		11 (1.8%)
None	238 (38.4%)	
Moderate	345 (55.6%)	
Severe	26 (4.2%)	
**Abdominal pain last month**		8 (1.3%)
No	498 (80.3%)	
Yes	114 (18,4%)	

* Alcohol Use Disorders Identification Test; Audit–C (risk consumption = women > 4, men > 5), [24].

** Saltin-Grimby – scale [25].

**Figure 2:**
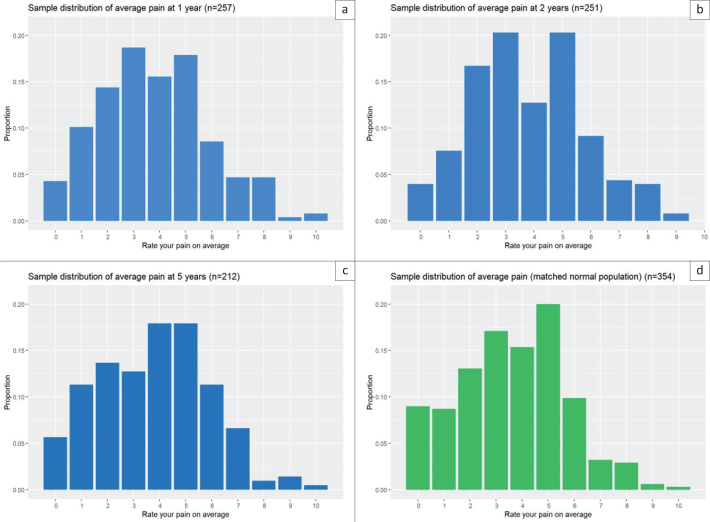
Average pain at the 1-, 2-, and 5-year follow-up and a matched reference population.

The number of respondents to the whole QoLiRECT questionnaire differs slightly from those included in the pain analysis, as a few participants did not provide responses to the BPI-SF. At the 5-year follow up, pain was prevalent in 37% (221/600) according to the BPI-SF. The corresponding prevalence was 269/854 (31%) at the 1-year follow-up and 258/764 (34%) at the 2-year follow-up. In the reference population the corresponding figure was 327/612 (53%). The level of average pain at 5 years was 3.8 (range 0–10) in the QoLiRECT cohort, a value that remained consistent at both the 1- and 2-year follow-ups. The average pain level in the reference population was comparable to that observed in the QoLiRECT cohort (3.6 (range 0–10)). The level of average pain at 1, 2, and 5 years as well as in the reference population are presented in Figure 2.

**Figure 3:**
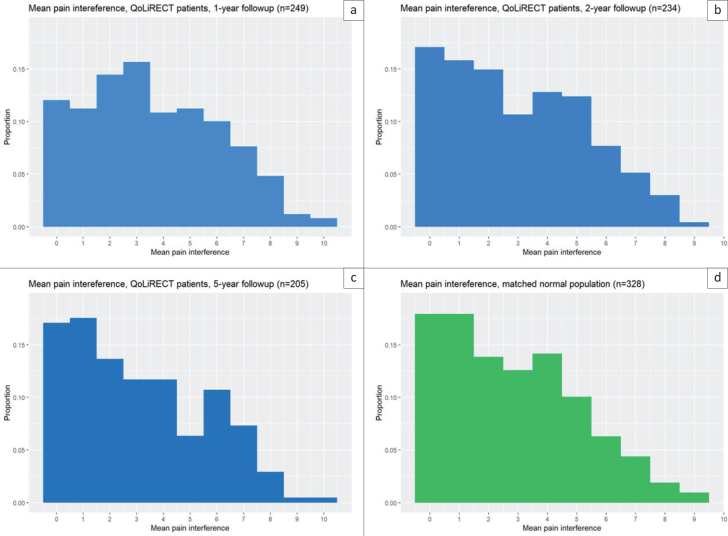
Interference of pain.

Regarding pain-related interference with daily life, only small differences were observed between the time points in the study cohort. The highest interferences were reported at 1 year, with a mean value of 3.6 (range 0–10). The corresponding figures at the 2- and 5-year follow-ups were only slightly lower, at 3.0 and 3.1. At 5 years, there was no statistical difference in mean pain-related interference between the cohort and the reference population (mean 3.0) (Figure 3).

**Figure 4:**
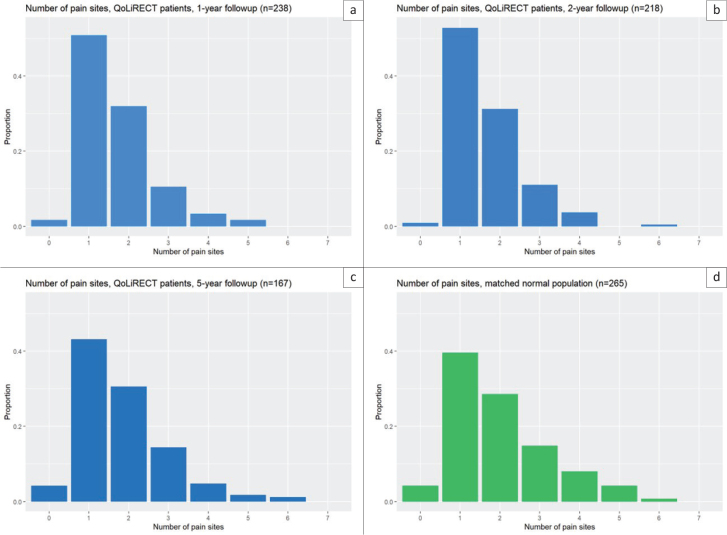
Number of pain sites.

There was no statistical difference in the number of pain sites between the cohort and the reference population even though there was a higher prevalence of pain localizations in the reference population compared to the QoLiRECT cohort. At the 5-year follow-up the mean number of reported pain sites was 1.8, compared to 1.7 at both the 1- and 2-year follow-up. In the reference population, the corresponding number was 2.0. The group reporting only one pain site was the largest at each follow-up point, as well as in the reference population (Figure 4).

At the 5-year follow-up, 600 patients out of the 620 who had responded to the QoLiRECT questionnaire could be analyzed according to the BPI-SF. Prevalence of pain was 36.8% (221/600). Among them pain in the lower extremities was prevalent in 19.3% (116/600), back pain 14.8% (89/600), pelvic pain 10.8% (65/600) and upper extremities 0.5% (63/600). Only 5.1% (31/600) had abdominal pain. There was a low prevalence of pain from the neck (2.8%, (17/600)) as well as headache (1.5%) (9/600)). Corresponding numbers in the reference group were 53.1% (329/614), 29.6% (182/614), 26.2% (161/614), 11.2% (69/614), 17.7% (109/614), 4.7% (29/614), 7% (43/614) and 4.6% (28/614), which indicates a minor difference, and a somewhat more widespread pain in the reference group than in the study group.

Three significant predictors of pain intensity 5 years after rectal cancer diagnosis were identified. Women had a greater risk of experiencing higher average pain (*p* = 0.004). Severe pain according to the pain item in the EQ-5D at baseline was also associated with higher risk of average pain (*p* = 0.036) as was higher BMI (*p* = 0.017). Results are presented in Table 3.

**Table 3:** Risk factors for average pain 5 years after curative treatment for rectal cancer.

Covariate	Estimate	CI (95%)	*P*
(Intercept**)**	5.38	1.429; 9.341	0.008
Age	-0.01	-0.003; 0.022	0.689
**Sex (male)**	-0.79	-1.331; -0.248	0.004
Type of surgery (APR)**	-0.22	-0.763; 0.327	0.432
**Type of surgery (Hartmann)**	0	-1.03; 1.023	0.994
Type of surgery (Other)	0.16	-1.736; 2.06	0.866
Preoperative radiotherapy	-0.42	-1.306; 0.462	0.347
Preoperative chemo/chemoradiotherapy	-0.43	-1.372; 0.518	0.374
Postoperative chemotherapy	0.05	-0.644; 0.748	0.883
UICC I	-0.79	-2.217; 0.629	0.273
UICC II	-0.79	-2.238; 0.665	0.287
UICC III	-0.54	-2.012; 0.928	0.468
UICC IV	-0.28	-1.948; 1.384	0.739
Depression (yes/maybe)	-0.58	-1.349; 0.193	0.141
**EQ-5D pain at baseline (moderate)**	0.3	-0.26; 0.851	0.296
**EQ-5D pain at baseline (severe)**	1.34	0.02; 2.658	0.047
**BMI**	0.07	0.008; 0.13	0.026
Not in a relationship	-0.46	-1.046; 0.131	0.127
University education	-0.16	-0.736; 0.421	0.591
ASA II	0.38	-0.258; 1.013	0.242
ASA III/IV	0.51	-0.414; 1.432	0.278
**Risk drinker (AUDIT-C)**	-0.32	-0.931; 0.29	0.302
**Physically active (Saltin-Grimby III–IV)**	-0.14	-0.967; 0.689	0.741
Sense of Coherence (KASAM)	-0.01	-0.029; 0.003	0.109
Time (2-year)	0.21	-0.182; 0.612	0.287
Time (5-year)	0.2	-0.279; 0.679	0.412

ASA: American Association of Anesthesiology.

## Discussion and conclusion

In this study, we found that patients who survived rectal cancer for at least 5 years after treatment reported less pain than a reference population without a history of rectal cancer. The experience of pain did not appear to change over time, as the level of average pain at 5 years was essentially identical to that at 1 and 2 years, and only minor changes were observed in terms of interference. Only small differences in both pain intensity and interference were observed compared to the reference population. Also, pain appeared to be somewhat more widespread throughout the body in the reference population.

Several previous studies have focused on assessing pain in expected locations such as the abdomen, pelvis and lower extremities after treatment for rectal cancer. Cancer pain is a complex condition [10], driven by inflammatory, neuropathic and cancer-specific mechanisms , and cancer treatment itself may also induce pain, for example, due to insufficiency fractures [13]. In this study, we chose to investigate pain with broader localization to facilitate comparison to a reference population. Our study is in accordance with previous research indicating that 30–50% of the patients experience pain 3 years after treatment for rectal cancer [7, 8, 23]. However, these studies have not compared data with a reference cohort, but in this study, we could see that prevalence of self-reported pain was similar, or even higher, in a reference population. The proportion of individuals indicating pain localized to the lower extremities was unexpectedly higher in the reference population than among the patients, and pain in the pelvic region was comparable. However, the fact that 40% of patients did not receive neoadjuvant treatment may explain why a large cohort did not have much pain in the pelvic area. Neoadjuvant treatment has been suggested as a risk factor for pelvic pain [39], which is also supported by data indicating that 10% of patients have an insufficiency fracture after neoadjuvant treatment [13]. Differences in neoadjuvant (chemo) radiotherapy strategies over time may also influence changes in pain localization and characteristics.

There are studies that have indicated that APE could increase the risk for pain compared to anterior resection [7] however, this was not replicated in a more recent cohort [39]. Thus, it remains uncertain what importance it has that only 33% underwent APE in our cohort.

In a Norwegian study comparing outcomes with a reference population, 25.4% reported chronic pain 1 year after curative treatment for rectal cancer, slightly lower than in our cohort. Chronic pain was associated with poorer functioning and lower healthrelated QoL [40]. The study is, however, not fully comparable to ours due to a slightly lower response rate, the use of a different pain assessment tool, and the fact that 10% of the cohort developed metastases, which may have influenced the results.

The high prevalence of pain among a random Swedish cohort could be considered somewhat surprising, as it suggests that half of the Swedish population experiences pain on a regular basis. However, there are previous studies supporting such numbers [41, 42]. Still in a previous study from Denmark chronic pain was prevalent in almost 30% of patients, which is less than in our cohort [43]. However, the use of dichotomous questions on chronic pain over the last 6 months compared to our study, which attempt to measure clinically relevant pain during the last week, may explain this difference. It is of course very difficult to truly explain the prevalence of pain as many other factors that may be difficult to measure affect pain*.* Psychosocial factors are important and the presence of pain is experienced and interpreted differently by individuals depending on other factors such as previous experiences in life and distress [44, 45].

It is possible that the prevalence of pain and the levels of average pain reported by patients surviving rectal cancer in our study are subject to a response shift, whereby patients after a life-threatening disease, such as cancer, alter their internal standards, values, or conceptualization of pain over time [46]. It is therefore possible that patients surviving rectal cancer do not perceive pain in the same way as individuals without a history of cancer, which could result in a more subdued perception of pain in the cancer group. However, it is of course not certain that this response shift is translated into a lower pain threshold. Future studies should focus on exploring pain in further detail to understand the underlying mechanisms. It is interesting that the cohort who had survived rectal cancer treatment did not experience a greater impact on daily life than the reference cohort. One might otherwise suspect that patients with a history of cancer would associate their pain with the treated cancer and therefore experience greater anxiety and impact on daily life.

The finding that patients reporting severe pain according to the EQ-5D pain question at baseline, as well as patients with overweight (measured with BMI) and of female sex, had an increased risk for high pain intensity at 5 years is important. It indicates that these patients could be targeted for interventions, perhaps through the administration of adjuvant analgesics to avoid long-term opioid consumption [47, 48]. The increased risk of pain associated with female sex, which is consistent with previous reports [6, 7, 24], may be difficult to counteract. In contrast, the higher risk observed in patients reporting pain at baseline, also reported in earlier studies [49, 50], highlights the need for careful follow-up regarding pain early in the post-treatment period, as chronic pain is difficult to treat [51]. It is possible that mitigation of pain in patients surviving rectal cancer could be achieved through increased exercise, as it has been suggested to reduce pain in patients with breast cancer; however, this remains to be studied [52].

The impact of pain on patients with cancer has been studied previously and has been shown to vary by cancer type, with higher pain levels prevalent in patients with more advanced disease, recent diagnoses, and recent cancer treatment [42]. We also observe that various instruments were used across studies assessing chronic and persistent pain, which may pose challenges when interpreting the results.

The strengths of this study include its multicenter design and the longitudinal follow-up, which enable comparisons over time. Another strength is the evaluation of non-included patients [26], which strengthens the extrapolation of our results. One limitation of this study is that the BPI-SF does not fully capture how frequently patients experience pain. It has previously been reported that a high frequency of pain negatively affects QoL [7]. Low-intensity pain occurring frequently may be comparable to, or even more harmful than, higher average pain levels in terms of its impact on QoL.

Another limitation is that we do not have baseline assessments of pain according to BPI-SF, since this instrument was not included in the baseline questionnaire. Several studies have indicated that pain experienced prior to or at the time of diagnosis is a risk factor for long-term pain [50], which is also supported by the findings in this study, where baseline pain according to EQ-5D was a significant risk factor. Also, it is possible that detailed information on pain at baseline could be flawed as patients are newly diagnosed with cancer. In addition, using a reference population could somewhat compensate for this. An additional limitation is the declining response rate over time, observed in both the QoLiRECT study and the reference-cohort. The patients were included approximately 10 years ago, and current treatments may therefore influence the pain experience to some extent. However, follow-up was completed in 2020, and the data can still be considered relatively recent.

In conclusion, we found that prevalence of pain is common in patients surviving rectal cancer, but it is even more common in a reference population, indicating that it is important to relate results from patients surviving cancer to a reference population. It is possible that pain after cancer treatment should be considered in the context of response shift, and some cancer survivors may perceive pain as less intense compared to a general population. Nevertheless, pain is often regarded as an important aspect of survivorship, and this type of questions merits further investigation.

## Data Availability

Request to access original data should be made to the corresponding author and will be considered based on context of the request.
